# Granulosa cell tumor in Arabian mares: prevalence, risk factors, clinical and histopathological findings and outcome of surgical removal

**DOI:** 10.3389/fvets.2025.1689782

**Published:** 2025-11-13

**Authors:** Derar Derar, Ahmed Ali, Fahd Al-Sobayil, Walid Refaai

**Affiliations:** 1 Department of Clinical Sciences, College of Veterinary Medicine, Qassim University, Buraydah, Saudi Arabia; 2 Department of Surgery, Anesthesiology and Radiology, University Veterinary Hospital, Qassim University, Buraidah, Saudi Arabia; 3 Department of Surgery, Anesthesiology and Radiology, Faculty of Veterinary Medicine, Zagazig University, Zagazig-City, El Sharkia, Egypt

**Keywords:** Arabian mare, tumor, complication, pregnancy, risk assessment

## Abstract

**Introduction:**

Granulosa cell tumors (GCTs) represent the most common ovarian neoplasms in mares, yet data on their epidemiology, risk factors, and clinical outcomes in Arabian horses are limited. Understanding their prevalence and diagnostic features is crucial for improving fertility management in this breed.

**Methods:**

Clinical records from 807 Arabian mares housed on 35 stud farms in central Saudi Arabia were retrospectively reviewed to determine the prevalence of GCT and potential risk factors, including age, parity, and anabolic steroid use. Twenty-four confirmed cases were further investigated using clinical examination, ultrasonography, endocrine profiling, and histopathology. Surgical management by unilateral ovariectomy was performed in 20 mares, and postoperative fertility outcomes were documented.

**Results:**

The overall prevalence of GCT was 0.6%. Logistic regression analysis identified anabolic steroid use as a significant risk factor (Odds Ratio = 13.21, *p* = 0.0001). Stallion-like behavior was the most frequent clinical manifestation (58.3%), followed by anestrus (33.3%) and persistent estrus (8.3%). Ultrasonography revealed four distinct tumor morphologies, with contralateral ovarian atrophy present in 75% of cases. Histopathology classified tumors into adult type (76.5%) and juvenile type (23.5%). Preoperative testosterone concentrations were elevated and decreased postoperatively in 72.7% of mares. Ovariectomy resulted in an 80% recovery rate, with 68.8% of mares regaining fertility.

**Discussion:**

This study demonstrates that anabolic steroid administration markedly increases the risk of GCT in Arabian mares. Behavioral changes, ultrasonographic appearance, and hormonal profiles remain key diagnostic indicators, while histopathology confirms tumor classification. Surgical excision proved effective, with favorable fertility outcomes in most mares. These findings underscore the importance of early diagnosis and highlight the need for judicious use of anabolic steroids in breeding programs.

## 1 Introduction

Granulosa cell tumors (GCTs) account for the majority of functional ovarian neoplasms in mares, making them the most commonly diagnosed reproductive tumor in this species (1–5). Although GCTs are generally benign and non-metastatic, they can cause profound reproductive dysfunction, including infertility, abnormal estrous cycles, and marked behavioral changes (6, 7). The neoplasm arises from granulosa and theca cells, and its endocrine activity often results in the excessive secretion of testosterone, inhibin, and anti-Müllerian hormone (AMH), which suppress contralateral ovarian function and contribute to clinical manifestations (8–11).

Clinically, affected mares may exhibit prolonged anestrus, persistent estrus, or stallion-like behavior, with the latter strongly associated with androgen overproduction (2, 12, 13). Ultrasonography has emerged as a cornerstone diagnostic tool, revealing pathognomonic morphologic patterns such as the multicystic “honeycomb” structure, solid masses, or mixed echotextures (14, 15). Hormonal assays, particularly testosterone and AMH measurement, have further improved diagnostic accuracy, enabling differentiation of GCTs from other ovarian conditions (16, 17). Histopathological evaluation remains essential for definitive diagnosis, with adult and juvenile variants distinguished by cellular morphology and mitotic activity (18, 19).

Although GCTs have been well-studied in other breeds, epidemiological data specific to Arabian mares-especially in Middle Eastern populations remain scarce. Moreover, potential risk factors such as anabolic steroid administration, widely recognized as an endocrine disruptor in both human and veterinary medicine, have received little empirical evaluation in the equine context (2, 20, 21). Given the breed’s economic and genetic value in the Arabian Peninsula, understanding the prevalence, risk factors, and clinicopathological characteristics of GCT in Arabian mares is essential for optimizing reproductive management and preserving breeding potential.

This study sought to estimate GCT prevalence in Arabian mares from Saudi Arabia, assess risk factors, particularly anabolic steroid exposure, characterize behavioral, ultrasonographic, and histopathological features, and analyze post-surgical fertility outcomes.

## 2 Materials and methods

### 2.1 Animals

A total of 807 Arabian mares from 35 stud farms in central Saudi Arabia were examined to determine the prevalence of granulosa cell tumors (GCT) and to investigate possible risk factors. For each mare, data on age, parity, and history of anabolic steroid administration were collected. Twenty-four mares were diagnosed with GCT based on a combination of clinical history, physical and reproductive examination, transrectal ultrasonography, gross pathological features of excised ovaries, and histopathological findings. Behavioral abnormalities-such as prolonged anestrus, persistent estrus, or stallion-like behavior-were documented through owner reports and direct observation.

### 2.2 Clinical examination

All mares underwent a general clinical assessment, including body condition scoring and measurement of vital parameters. Reproductive tract evaluation consisted of rectal palpation and ultrasonography using a 5 MHz linear transducer (Aloka SSD-500, Aloka Co., Ltd., Tokyo, Japan). Tumor characteristics including size (length, width, height), echotexture (homogeneous or heterogeneous), internal architecture, presence of cystic or follicular structures, and contralateral ovarian activity were recorded.

### 2.3 Surgical ovariectomy

Unilateral ovariectomy was performed in 20 standing sedated GCT mares under strict aseptic conditions. Sedation was induced with detomidine hydrochloride (0.01–0.02 mg/kg IV) combined with butorphanol tartrate (0.01 mg/kg IV), followed by local anesthesia using an inverted-L block at the paralumbar fossa with 2% lidocaine. A vertical or slightly oblique flank incision was made through the skin and abdominal musculature to expose the peritoneum, which was then incised to access the ovary. The enlarged ovary was exteriorized, and the ovarian pedicle was ligated using absorbable sutures or sealed with a vessel-sealing device to control hemorrhage. The affected ovary was removed en bloc, and the pedicle was inspected for hemostasis before being returned into the abdominal cavity. Abdominal wall layers were closed with absorbable sutures, and the skin was sutured with non-absorbable material. Postoperative management included broad-spectrum antimicrobial therapy, non-steroidal anti-inflammatory drugs (A combination of Procaine/penicillin G 22,000 IU/kg IM, Gentamicin 6.6 mg/kg IV and Flunixin meglumine 1.1 mg/kg IV daily for 5 days) and daily wound inspection for early detection of complications. Owners were advised to restrict activity for 14 days post-surgery.

### 2.4 Histopathology

Out of the 24 GCT-mares in this study, 20 unilaterally-GCT affected mares were surgically ovariectomized; ovarian specimens obtained from 17 mares for histopathological examination. Specimens were fixed in 10% neutral buffered formalin, dehydrated through graded ethanol, cleared in xylene, and embedded in paraffin. Sections of 2–5 μm thickness were stained with hematoxylin and eosin (22). Microscopic examination included assessment of growth pattern, cellular morphology, nuclear features, mitotic count per high-power field (HPF), stromal component, and presence of diagnostic structures such as Call-Exner bodies.

### 2.5 Hormonal assay

Blood samples were collected via jugular venipuncture just before surgery and again 24 h post-operatively. Serum was separated by centrifugation at 1,200 × g for 10 min and stored at −20 °C. Testosterone concentrations were measured using a commercial ELISA kit (Immunocentrix, Canoga Park, CA, USA). Intra- and inter-assay coefficients of variation were 15.7 and 8.5%, respectively.

### 2.6 Statistical analysis

Data were expressed as mean ± standard error of the mean (SEM). Binary logistic regression was applied to determine the association between GCT occurrence and potential risk factors. Differences in testosterone levels between the adult and juvenile types were analyzed using a *t*-test. Statistical analyses were performed using SPSS version 24.0 (IBM Corp., Chicago, IL, USA), and significance was set at *p* < 0.05.

## 3 Results

### 3.1 Prevalence and risk factors

GCT was diagnosed in 24 of the 807 mares, giving a prevalence of 0.6%. Logistic regression revealed a significant association between anabolic steroid use (Nandrolone laurate 1 mg/kg ranged from a single dose to repeated injections at 1–3 week intervals and/or testosterone propionate 50 mg IM once weekly) and GCT occurrence [Odds ratio (OR) = 13.21, *p* = 0.0001], whereas age and parity were not significant predictors (Table 1).

**Table 1:** Risks associated with GCT in Arabian mares.

Item	*B*	S.E.	Wald	df	Sig.	Exp (B)	95% C.I. for EXP(B)	
							Lower	Upper
Steroids	2.171	0.597	13.208	1	0.000	8.768	2.719	28.275
Age	−0.012	0.515	0.001	1	0.982	0.988	0.360	2.709
Parity	0.251	0.522	0.232	1	0.630	1.285	0.463	3.572
Constant	−2.798	1.109	6.363	1	0.012	0.061		

### 3.2 Breeding and Behavioral history

Of the affected mares, 54.16% were under 10 years of age, 66.66% were multiparous, and 58.33% had a documented history of anabolic steroid use (Table 2). Stallion-like behavior was the most frequent clinical sign (58.33%).

**Table 2:** Breeding history of Arabian mares with GCT.

Item		Number of mare	%
Age	<10 years	13	54.16%
	>10 years	11	45.83%
Parity	Nullipara	8	33.33%
	Multipara	16	66.66%
Use of anabolic steroids	Yes	14	58.33%
	No	10	41.66%
Behavior	Anestrus	8	33.33%
	Male behavior	14	58.33%
	Nymphomaniac	2	8.34%

### 3.3 Clinical and ultrasonographic findings

**Figure 1:**
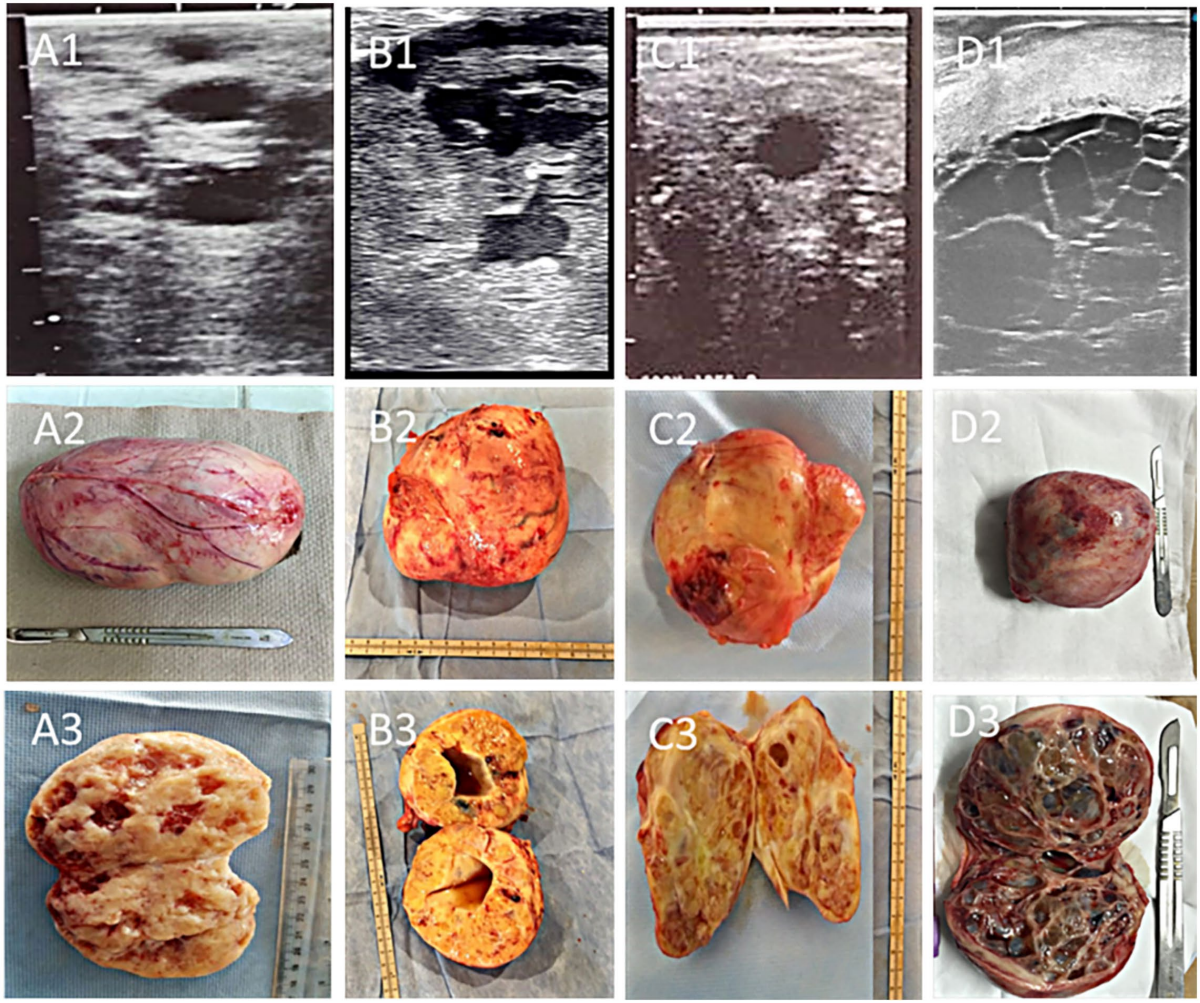
Ultrasonographic, gross, and cross-sectional appearance of granulosa cell tumors (GCT) in mares: **(A1–A3)** compact GCT with multiple scattered cysts, showing a hypoechoic mass with numerous anechoic cavities separated by echogenic septa; **(B1–B3)** single-cavitary solid GCT, characterized by a large central anechoic cavity surrounded by solid echogenic tissue; **(C1–C3)** homogenous compact GCT with only a few small cystic foci; and **(D1–D3)** multilocular honeycomb-organized GCT with multiple adjacent anechoic cavities divided by fine echogenic septa. Cystic structures in the first three forms generally contain serosanguinous fluid, whereas the honeycomb type is typically filled with hemorrhagic contents.

Tumors ranged in size from 9 × 7 × 8 cm to 17 × 14 × 12 cm (average 13.53 ± 0.67 × 11.31 ± 0.50 × 8.99 ± 0.38 cm). The left ovary was affected in 13/24 (54.16%) of cases, the right in 10/24 (41.6%) of cases, and both ovaries in one (4.16%) case. Four distinct ultrasonographic and gross pathological patterns were recognized (Figure 1): compact masses with numerous small cysts (6 cases), single large solid-cavituous masses (2 cases), homogeneous solid masses with few small cysts (7 cases), and multilocular “honeycomb” cystic structures with hemorrhagic fluid (9 cases). Follicular activity was absent in 87.5% of affected ovaries, and contralateral ovarian atrophy was present in 75% of mares.

### 3.4 Gross and histopathological findings

**Figure 2:**
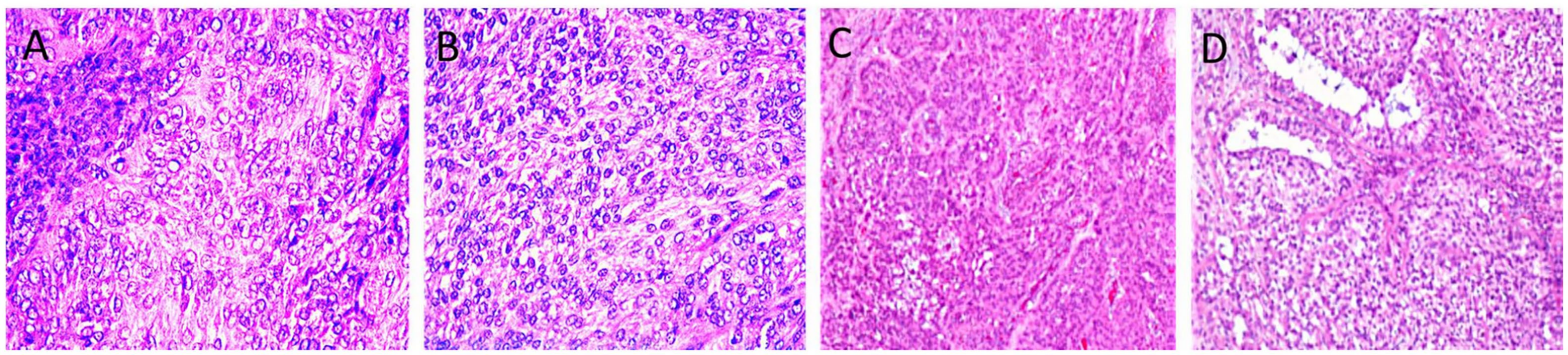
Ovarian tissues with adult type **(A, B)** and juvenile type granulosa cell tumor **(C, D)**. Note the necrotized nuclei with some area of calcification (H&E X400, **A**). Insular pattern of the polyhedral neoplastic cells with numerous mitotic figures (H&E, X400, **B**). Lesion exhibiting sheets and clusters of polygonal cells having coffee bean nuclei and occasional prominent nucleoli (H&E X40, **C**). Trabecular and microfollicular patterns of tumor cells are also noted. Focally myxoid material is seen in the follicles Brownish pigment is also noted (H&E X40, **D**).

Of the 17 histopathologically confirmed cases, 13 were adult-type and 4 were juvenile-type GCTs (Figure 2). Grossly, adult-type GCT are typically characterized by firm, lobulated masses with tan-yellow cut surfaces and cystic cavities that are filled with serosanguinous fluid. Microscopically, diffuse to lobular arrangements of polyhedral granulosa cells with eosinophilic cytoplasm, round-to-oval nuclei, and occasional Call-Exner bodies were observed. Mitotic activity ranged from 1 to 3/HPF, with focal hemorrhage and necrosis. Juvenile-type GCTs typically demonstrate a softer texture and a more homogeneous cut surface, with negligible cystic alteration. Histologically, this type exhibits sheets of uniform small cells with scant cytoplasm, a high nuclear-to-cytoplasmic ratio, and frequent mitotic Figures (5-8/HPF). Follicular structures were uncommon, and stromal tissue was sparse.

### 3.5 Surgical outcomes

Of the 20 mares that underwent unilateral ovariectomy, 16/20 mares (80%) recovered. Four mares (20%) developed postoperative complications, including incisional infection and hemorrhage. Sixteen mares returned to breeding; the first estrus occurred within 1–4 months (2.3 ± 0.7 months), and conception occurred within 3–14 months (6.7 ± 2.9 months). The overall conception rate was 68.75% in mares bred post-surgery.

### 3.6 Hormonal profiles

In adult-type GCTs, preoperative testosterone levels ranged from 4.3 to 18.25 ng/mL (13.2 ± 3.4 ng/mL), while juvenile-type tumors showed a wider range from 0.01 to 21.64 ng/mL (11.25 ± 4.12 ng/mL). Difference between adult and juvenile types was significant (*p* = 0.001). Postoperative measurements revealed a decline in testosterone concentrations in 72.7% of cases, while 27.3% showed no significant change within 24 h after surgery.

## 4 Discussion

To the authors’ knowledge, this is the first comprehensive epidemiological and clinicopathological study of granulosa cell tumors (GCT) in Arabian mares in Saudi Arabia. The observed prevalence of 0.6% among 807 mares aligns with earlier reports indicating that GCTs, while the most common ovarian neoplasm in the mare, remain relatively rare in the general population (1, 4, 23). Previous large-scale surveys in different geographical contexts have reported prevalence values ranging from 0.4 to 2% depending on the population studied and the diagnostic criteria employed (6, 24). The variation may reflect differences in breed predisposition, management practices, and regional veterinary diagnostic capacities.

Notably, anabolic steroid administration was strongly linked to GCT development, increasing the likelihood of tumor occurrence by a factor of 13.21. While previous equine literature has not quantified this relationship to this extent, anabolic steroid administration has been recognized as an endocrine disruptor capable of altering ovarian steroidogenesis and gonadotropin feedback mechanisms (7, 21). Chronic exogenous androgen exposure could potentially promote granulosa-theca cell proliferation via upregulation of growth factors and suppression of follicular atresia, thereby increasing neoplastic risk (2, 9, 20). Comparable mechanisms have been postulated in human and rodent models, where anabolic steroid exposure has been implicated in ovarian stromal hyperplasia and tumorigenesis (20). The present results therefore contribute novel quantitative evidence to support caution in the non-therapeutic use of anabolic steroids in mares.

In this study, stallion-like behavior was the most common behavioral change (58.33%), followed by anestrus (33.33%) and continuous estrus (8.34%). This pattern is consistent with the literature, in which virilization is a hallmark of hormonally active GCTs, often linked to elevated testosterone secretion by the tumor (6, 25). Stallion-like behavior may include aggression, mounting, and vocalization, reflecting androgenic effects on the central nervous system (13). However, not all mares with GCT exhibit behavioral changes, and not all behavioral alterations correlate with elevated androgens (3). This underscores the importance of integrating hormonal and imaging diagnostics rather than relying solely on behavior for suspicion of GCT.

The ultrasonographic appearance of GCT in this cohort exhibited considerable variation, including compact forms with multiple cysts, single-cavity solid masses, homogeneous masses with few cysts, and multilocular “honeycomb” masses filled with hemorrhagic fluid. These observations concur with earlier descriptions, where the “honeycomb” and “solid” patterns are most commonly reported (14, 15). The absence of follicular activity in the affected ovary and contralateral ovarian atrophy also corroborate the well-documented endocrine suppression exerted by GCTs via excessive production of testosterone, inhibin and anti-Müllerian hormone (3, 10, 16, 26). The ultrasonographic identification of such characteristic patterns remains a cornerstone in the presumptive diagnosis of GCT in mares.

The present study confirms the existence of both adult and juvenile granulosa cell tumor (GCT) histotypes in Arabian mares, each with distinct morphological characteristics and prognostic relevance. Adult-type GCTs predominantly affect middle-aged to older mares and generally exhibit indolent growth, while juvenile-type tumors, though less frequently described in equines, tend to occur in younger mares and can display more rapid enlargement, reflecting patterns previously reported in the literature (3, 17, 18, 23, 27, 28). Recent equine-specific immunohistochemical data highlight several promising diagnostic markers: in a pilot equine study, granulosa cells of a GCT demonstrated pronounced membranous staining for moesin and phosphorylated ezrin (p-ezrin), along with a low Ki-67 proliferation index-findings that support the generally benign behavior of these tumors (29, 30). Additional markers-including E-cadherin, calretinin, AMH, and aromatase-have shown potential in differentiating cellular components within equine sex cord-stromal tumors and improving tumor classification (11, 29).

In human GCTs, adult-type tumors frequently harbor the FOXL2 C134W mutation, which is considered pathognomonic and implicated in granulosa cell differentiation and tumorigenesis (31). Although the presence and role of FOXL2 mutations in equine GCTs remain unexplored, FOXL2’s central function in granulosa cell development suggests that investigating this pathway in horses could yield valuable insights.

From a clinical perspective, accurate histological and molecular subtyping bears significant implications. Adult GCTs-with their typical endocrine profiles and benign course-may warrant standard postoperative surveillance, focusing on hormone normalization. Conversely, juvenile-type GCTs may necessitate more vigilant follow-up due to their potential for rapid growth and distinct hormonal activity. Expanding studies to investigate ERM proteins, AMH/inhibin dynamics, proliferation indices, and FOXL2 status across tumor subtypes holds promise for enhancing prognostic stratification and informing tailored postoperative management in equine practice.

Preoperative testosterone concentrations in adult-type GCTs in the present study were elevated compared to physiological levels (<0.2 ng/mL), aligning with prior findings (3, 12, 32). The wide range observed in juvenile types reflects the heterogeneity of steroidogenic activity among tumors. Postoperative declines in testosterone mirror the expected endocrine resolution following tumor excision (10, 26, 28), although persistent elevation in a subset may be attributable to residual tumor tissue, delayed contralateral ovarian recovery, or concurrent endocrine disorders. Postoperative hormone sampling in this study was performed at 24 h after unilateral ovariectomy. Although testosterone concentrations declined in the majority of mares, 27.3% showed no significant change at this early time point. This finding is consistent with previous reports showing variable time courses for biochemical normalization after removal of granulosa-cell tumors. Testosterone normalization may take from days to several weeks or months in individual mares (15, 30). Therefore, a single 24-h postoperative sample may underestimate the proportion of mares that will ultimately achieve hormonal normalization; serial sampling (weekly or every 2 weeks) would provide a more definitive assessment.

The recovery rate following unilateral ovariectomy in this study is within the upper range of previously reported outcomes (33, 34). Furthermore, the postoperative conception rate of this study is comparable to those documented previously (34), supporting the premise that timely surgical intervention can restore fertility in a majority of cases. Complications highlight the inherent risks of large ovarian mass removal, particularly in cases complicated by hemorrhage or adhesions (35–37). Minimally invasive techniques, such as laparoscopic-assisted removal, have been advocated to reduce complications in suitable cases (38).

The pathogenesis of GCT in mares likely involves a complex interplay of genetic, endocrine, and environmental factors (17, 23). Dysregulation of granulosa cell proliferation may result from aberrant activation of signaling pathways involved in folliculogenesis, such as the TGF-*β* superfamily (9). Testosterone, Inhibin and anti-Müllerian hormone overproduction suppress contralateral ovarian function, accounting for the frequent anovulation of the unaffected ovary (10, 16, 26). The novel association identified in this study between anabolic steroid exposure and GCT occurrence may reflect direct mitogenic effects of exogenous androgens on ovarian stromal and granulosa cells, or indirect effects via hypothalamic–pituitary-gonadal axis modulation (20, 21).

The primary limitations of this study include the relatively small number of GCT cases, which may constrain the precision of prevalence and risk factor estimates, and the geographic restriction to central Saudi Arabia, which may limit generalizability to other regions or breeds. Additionally, while the association with anabolic steroid use was statistically significant, causality cannot be inferred from this observational design. The reliance on owner-reported steroid use also introduces the possibility of recall bias.

The findings underscore the necessity for judicious use of anabolic steroids in equine practice, particularly in breeding mares. Behavioral changes, especially stallion-like traits, should prompt ultrasonographic evaluation and hormonal profiling to facilitate early detection. Histopathological subtyping should be routinely performed to enhance prognostic accuracy. Surgical removal remains the treatment of choice, with a high likelihood of restoring fertility when undertaken early.

## 5 Conclusion

Granulosa cell tumor GCT poses significant reproductive and behavioral challenges especially in competitive and performer Arabian mares. The present study provides the first epidemiological evidence linking anabolic steroid use to a markedly increased risk of GCT in Arabian breed in Saudi Arabia. Characteristic ultrasonographic features, in conjunction with hormonal profiling, allow for reliable preoperative diagnosis. Surgical removal offers a high likelihood of recovery and restoration of fertility, particularly when performed promptly. The results underscore the necessity for prudent regulation of anabolic steroid use in equine breeding programs and highlight the value of routine reproductive screening for early detection and improved prognosis.

## Data Availability

The raw data supporting the conclusions of this article will be made available by the authors, without undue reservation.
